# Characterization of cardiometabolic risk awareness among patients with psoriasis: A quality improvement survey study

**DOI:** 10.1016/j.jdin.2024.03.020

**Published:** 2024-04-06

**Authors:** Caitlin A. Kearney, Sreejan Saha, Maria Teresa Mata Vivas, Joel M. Gelfand, Jessica Garelik, Kristen I. Lo Sicco, Michael Garshick

**Affiliations:** a New York University Grossman School of Medicine, New York, New York; b New York Institute of Technology College of Osteopathic Medicine, Old Westbury, New York; c Center for the Prevention of Cardiovascular Disease and Leon H. Charney Division of Cardiology, Department of Medicine, New York University Grossman School of Medicine, New York, New York; d Department of Dermatology, University of Pennsylvania Perelman School of Medicine, Philadelphia, Pennsylvania; e The Ronald O. Perelman Department of Dermatology, New York University Grossman School of Medicine, New York, New York

**Keywords:** cardiometabolic risk, cardiovascular disease, metabolic syndrome, psoriasis, psoriasis comorbidities, quality improvement

*To the Editor:* Psoriasis is associated with an elevated risk of cardiovascular disease (CVD).^1^ The American Academy of Dermatology-National Psoriasis Foundation guidelines of care for psoriasis recommend that dermatologists educate patients on their increased CVD risk and ensure appropriate screening.^2^ Despite this, patients with psoriasis have underrecognized and undertreated cardiometabolic risk factors.^3^ To inform the development of CVD risk reduction strategies, we undertook a quality improvement (QI) project to assess the awareness of cardiometabolic risk and receptiveness to further education among patients with psoriasis.

Patients with psoriasis receiving topical, systemic, or phototherapy treatment at the New York University Langone psoriasis specialty clinic or phototherapy clinic were approached between July 2022 and March 2023 as a convenience sample. An anonymous 47-question paper survey was administered after clinic visits to assess cardiometabolic medical history, risk screening, and awareness. Questions were modeled after the Centers for Disease Control 2021 to 2022 National Health and Nutrition Examination Survey.^4^ Multivariate logistic regression analyses were performed in RStudio (version 2023.06.0 + 421) to evaluate associations between clinical factors and CVD screening recommendations or awareness. IRB review was not required as this project satisfied the definition of QI.

Of the 112 patients approached, 101 completed the survey (90.2% response rate). Most participants reported that they saw a primary care provider within the past year (79 participants [78.2%]) and underwent evaluation of cardiometabolic risk factors, including blood pressure (99 [98.0%]), weight (100 [99.0%]), cholesterol (87 [86.1%]), and hemoglobin A1c (64 [63.4%]), within the past 2 years (Table I). Despite this, just 34 participants (33.7%) reported that the connection between psoriasis and CVD had been discussed with them. Male participants were more likely to report that screening for risk of CVD has been recommended (adjusted odds ratio 3.10, 95% CI [1.07, 9.95], *P* < .05) and the connection between psoriasis and CVD has been discussed (3.80 [1.36, 11.82], *P* < .05) in comparison to female participants (Table II). Most participants (84 [83.2%]) desire education regarding psoriasis and CVD and prefer this information from their dermatologist (73 [72.3%]).

**Table I:** Survey participant-reported cardiometabolic risk factor screening history, cardiometabolic comorbidities, prior education, and receptiveness to further education regarding psoriasis and cardiovascular disease

Variables (*N* = 101)	Study participants	
	No.	%
Primary care and specialists seen by participants within the last year		
Primary care provider	79	78.2
Cardiologist	36	35.6
Rheumatologist	32	31.7
Primary dermatologist∗	33	32.7
Cardiometabolic risk factor screening history		
Blood pressure		
Checked within the last 2 years	99	98.0
Not checked within the last 2 years	2	2.0
Do not know	0	0
Weight		
Checked within the last 2 years	100	99.0
Not checked within the last 2 years	1	1.0
Do not know	0	0
Blood cholesterol		
Checked within the last 2 years	87	86.1
Not checked within the last 2 years	5	5.0
Do not know	9	8.9
Hemoglobin A1c		
Checked within the last 2 years	64	63.4
Not checked within the last 2 years	7	6.9
Do not know	30	29.7
Participant-reported cardiometabolic risk factors		
High blood pressure	36	35.6
High cholesterol	48	47.5
Diabetes	15	14.9
Borderline or prediabetes	16	15.8
Overweight	47	46.5
Current smoker	6	5.9
Previous education on connection between psoriasis and CVD by doctor or health professional		
Connection discussed with participant	34	33.7
By primary dermatologist	9	8.9
By phototherapy clinic dermatologist	5	5.0
By psoriasis specialty clinic dermatologist	9	8.9
By primary care provider	11	10.9
By cardiologist	15	14.9
By rheumatologist	7	6.9
By other provider	4	4.0
Connection never discussed with participant	67	66.3
Receptiveness to further education on connection between psoriasis and CVD		
Receptive to further education	84	83.2
By dermatologist	73	72.3
By primary care provider	57	56.4
By preventative cardiologist	33	32.7
By rheumatologist	32	31.7
By nutritionist	21	20.8
Not receptive to further education	17	16.8

*CVD*, Cardiovascular disease.

∗ Primary dermatologist is defined as the referring dermatologist to our subspecialty clinics (phototherapy clinic or psoriasis specialty clinic) who is responsible for yearly skin checks and other general dermatology issues.

**Table II:** Associations between clinical factors and whether participants have been recommended to undergo screening for their risk of cardiovascular disease or have been educated on the connection between psoriasis and cardiovascular disease by a doctor or other health professional

Variable	*n*	Screening recommended		Aware of psoriasis/CVD connection	
		Adjusted odds ratio [95% CI]	*P* value	Adjusted odds ratio [95% CI]	*P* value
Location					
Phototherapy clinic	36	1.00 [ref]	-	1.00 [ref]	-
Psoriasis specialty clinic	65	0.96 [0.33, 2.85]	.95	1.35 [0.48, 3.94]	.57
Age					
18-49	36	1.00 [ref]	-	1.00 [ref]	-
50-69	38	4.19† [1.32, 14.88]	.02∗	1.51 [0.50, 4.72]	.47
70-89	27	2.11 [0.59, 7.97]	.26	2.03 [0.62. 6.96]	.25
Race					
White	65	1.00 [ref]	-	1.00 [ref]	-
Black	6	0.84 [0.09, 6.59]	.87	1.21 [0.14, 8.15]	.85
Other	30	0.51 [0.13, 1.77]	.31	0.82 [0.24, 2.71]	.76
Sex					
Female	41	1.00 [ref]	-	1.00 [ref]	-
Male	60	3.10‡ [1.07, 9.95]	.04∗	3.80‡ [1.36, 11.82]	.01∗
Ethnicity					
Hispanic	14	1.16 [0.23, 5.81]	.85	1.21 [0.26, 5.43]	.80
Primary care provider					
Seen within 1 year	79	1.65 [0.47, 6.54]	.45	0.66 [0.19, 2.22]	.49
Rheumatologist					
Seen within 1 year	32	0.78 [0.26, 2.21]	.64	1.73 [0.65. 4.72]	.28
Primary dermatologist					
Seen within 1 year	33	0.96 [0.30, 3.00]	.94	1.18 [0.38, 3.66]	.78

Multivariate logistic regression analyses were performed in RStudio (version 2023.06.0 + 421). Odds ratios are adjusted for other variables included in the multivariate logistic regression model. Significance (*P* < .05) is indicated with an asterisk.

*CVD*, Cardiovascular disease.

† OR adjusted for location, race, sex, ethnicity, primary care provider visit within 1 year, rheumatologist visit within 1 year, and primary dermatologist visit within 1 year.

‡ OR adjusted for location, age, race, ethnicity, primary care provider visit within 1 year, rheumatologist visit within 1 year, and primary dermatologist visit within 1 year.

Potential limitations include that this was a pilot QI project and, thus, due to a small sample size, is potentially underpowered. Other limitations are survey response subjectivity, self-report bias, and variable health literacy. As a QI study limited to New York University, this data are not generalizable.

Our findings suggest that patients with psoriasis have not received effective counseling regarding their elevated cardiometabolic risk, despite a high prevalence of recent primary care visits and screening tests. This study expands upon previous studies characterizing poor patient awareness by demonstrating a gender disparity in patient education and patient preference for education by dermatologists over other providers.^5^ As the primary providers of psoriasis management and patients’ preferred educators, dermatologists are uniquely situated to inform patients of the cardiometabolic risk associated with psoriasis and facilitate early screening. Potential barriers to counseling by dermatologists include lack of time during visits, knowledge of screening guidelines, and resources to manage screening results. Improved awareness of appropriate screening practices and interdisciplinary care coordination for the management of identified cardiometabolic risk factors could help dermatologists advance educational and screening efforts.

## Conflicts of interest

Dr Gelfand served as a consultant for AbbVie, Artax (DSMB), BMS, Boehringer Ingelheim, Celldex (DSMB), FIDE (which is sponsored by multiple pharmaceutical companies) GSK, Inmagene (DSMB), Twill, Lilly (DMC), Leo, Moonlake (DSMB), Janssen Biologics, Novartis Corp, UCB (DSMB), Neuroderm (DSMB), and Veolia North America receiving honoraria; and receives research grants (to the Trustees of the University of Pennsylvania) from Amgen and Pfizer Inc. Dr Lo Sicco is a current investigator for Pfizer Inc; former investigator for Regen; and consultant for Pfizer Inc and Acquis. Dr Garshick is a consultant for AbbVie and Horizon Therapeutics. Authors Kearney, Saha, Mata Vivas, and Garelik have no conflicts of interest to declare.

## References

[1] Masson W., Lobo M., Molinero G. (2020). Psoriasis and cardiovascular risk: a comprehensive review. Adv Ther.

[2] Elmets C.A., Leonardi C.L., Davis D.M.R. (2019). Joint AAD-NPF guidelines of care for the management and treatment of psoriasis with awareness and attention to comorbidities. J Am Acad Dermatol.

[3] Song W.B., Peck G.M., Neopaney A. (2023). Regional variation in cardiovascular risk factor screening by dermatologists for psoriasis patients in the United States. J Invest Dermatol.

[4] Centers for Disease Control and Prevention (CDC), National Center for Health Statistics (NCHS) (2022). https://wwwn.cdc.gov/nchs/nhanes/continuousnhanes/questionnaires.aspx?BeginYear=2021.

[5] Barbieri J.S., Beidas R.S., Gondo G.C. (2022). Analysis of specialist and patient perspectives on strategies to improve cardiovascular disease prevention among persons with psoriatic disease. JAMA Dermatol.

